# Penetration of the sigmoid colon to the posterior uterine wall secondary to diverticulitis: a case report

**DOI:** 10.4076/1752-1947-3-8957

**Published:** 2009-08-24

**Authors:** Tomoyuki Akiyama, Masahiko Inamori, Takeshi Shimamura, Hiroshi Iida, Hiroki Endo, Koji Fujita, Masato Yoneda, Hirokazu Takahashi, Yasunobu Abe, Noritoshi Kobayashi, Kensuke Kubota, Hiroshi Kobayashi, Shoji Yamanaka, Yasushi Rino, Atsushi Nakajima

**Affiliations:** 1 Gastroenterology Division, Yokohama City University Hospital, Yokohama, Japan; 2 Department of Internal Medicine and Clinical Immunology, Yokohama City University Hospital, Yokohama, Japan; 3 Division of Pathology, Yokohama City University Hospital, Yokohama, Japan; 4 Division of Surgery, Yokohama City University Hospital, Yokohama, Japan

## Abstract

**Introduction:**

Penetration of the colon to the posterior uterine wall secondary to diverticulitis is unusual, with diagnostic methods not yet established. Non-invasive imaging, such as computed tomography and magnetic resonance imaging may help to establish a proper diagnosis, but confirmation may be reached only after surgical exploration.

**Case presentation:**

We report the case of a 78-year-old Japanese woman who presented with a low grade fever and mild diarrhea which occurred two or three times a week. Computed tomography and magnetic resonance imaging demonstrated a capsular lesion including an air structure with a diameter of 5 cm, between the posterior aspect of the uterine body and the sigmoid colon. A gastrograffin enema and colonoscopy demonstrated a giant diverticulum of the sigmoid colon with no evidence of malignancy. These data confirmed the diagnosis of diverticulitis complicated by a giant diverticulum. Because of a relapsing fever after therapy with antibiotics, the patient had en bloc surgical treatment of the uterus, fallopian tubes, ovaries and sigmoid colon, the organs involved in the diverticulitis, followed by an uneventful recovery.

**Conclusion:**

This is a rare case report of penetration of the sigmoid colon to the posterior uterine wall secondary to diverticulitis.

## Introduction

Diverticulosis is the most common colonic disease. Up to 30% of individuals are affected by the time they reach 60 and nearly 65% by the age of 80 [1]. In this patient population, 25% will be complicated with diverticulitis, an inflammatory process that may require surgery for abscesses, hemorrhage, perforation, or fistula formation. Colovesical fistula formation is the most common, while colouterine fistula is an extremely rare disease due to the resistance of uterine tissue [2,3]. Penetration of the sigmoid colon to the wall of the uterus is considered as an early stage condition in the formation of a colouterine fistula. We report a case of a patient with penetration of the sigmoid colon to the posterior wall of the uterus secondary to diverticulitis.

## Case presentation

A 78-year-old Japanese woman, with a previous medical history of arterial hypertension and who had not undergone any previous operations, was admitted to our hospital with a low grade fever and mild diarrhea, which had occurred two or three times a week during the six months before admission. Physical examination revealed no spontaneous pain, no tenderness, and no guarding in the abdomen, and no abdominal or pelvic mass was present on palpation. The patient was afebrile, but there were mild inflammatory signs in the laboratory data (C-reactive protein: 1.1 mg/dL, white blood cells: 10,400/μL). The other biological values (hemoglobin, electrolytes, urine) were normal.

**Figure 1:**
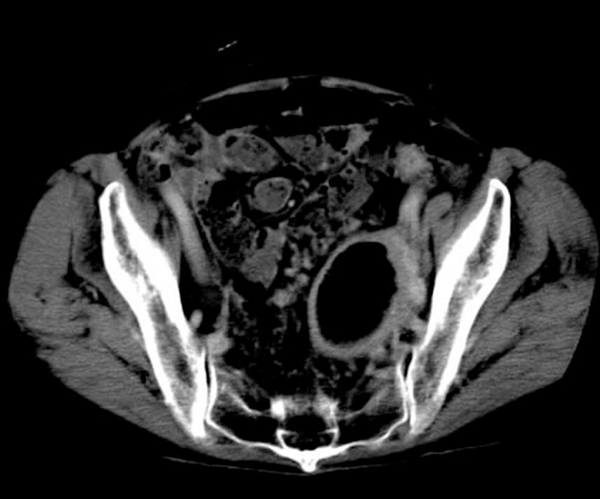
**Computed tomography revealed the capsular lesion including air density with a diameter of 5 cm between the posterior wall of the uterine body and the sigmoid colon**.

**Figure 2:**
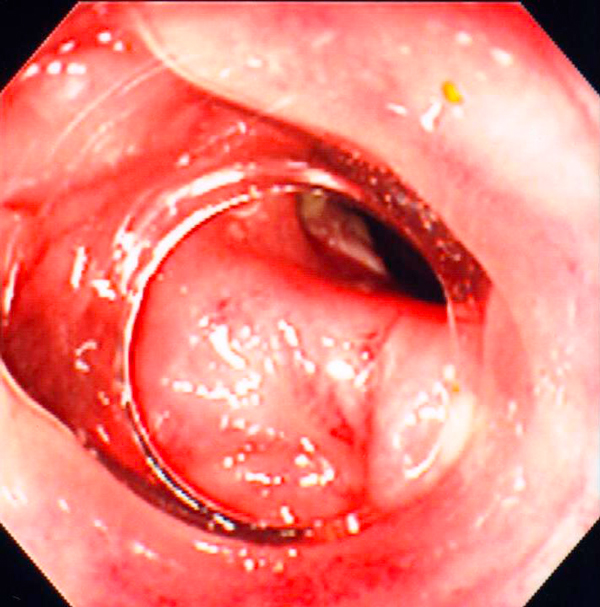
**Colonoscopy showed a giant diverticulum of the sigmoid colon without evidence of malignancy, penetrating to the wall of the uterus**.

**Figure 3:**
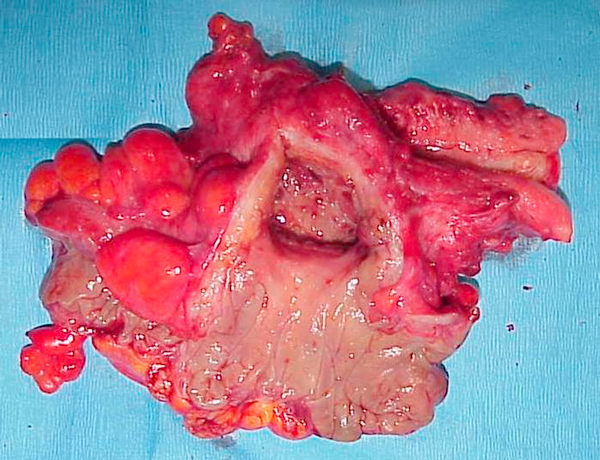
**Pathological examination confirmed a giant diverticulum with inflammation and abscess of the sigmoid colon, penetrating to the posterior wall of the uterus**.

First, we performed computed tomography (CT) and magnetic resonance imaging (MRI) as non-invasive imaging modalities. The CT scan revealed a capsular lesion including air density with a diameter of 5 cm between the posterior wall of the uterine body and the sigmoid colon (Figure 1). The MRI scan showed the capsular lesion including an air structure. A gastrograffin enema and colonoscopy demonstrated a giant diverticulum of the sigmoid colon without evidence of malignancy and penetration to the wall of the uterus (Figure 2). These data confirmed the diagnosis of diverticulitis complicated by a giant diverticulum. Since these clinical manifestations, imaging findings and colon examinations did not suggest the presence of any severe complications, such as a penetration of the sigmoid colon to the posterior uterine wall, sigmoid-uterine fistula, perforation, or peritonitis, we selected a conservative therapy. Therapy with antibiotics was instituted for two weeks, and although improvement in the low grade fever was observed, the fever redeveloped. We proceeded to laparotomy where we found a portion of the sigmoid colon was adherent to the uterine fundus. An en bloc resection of the sigmoid colon with the uterus and adnexae was performed, as well as a side-to-end colorectal anastomosis. Pathological examination confirmed a giant diverticulum with inflammation and abscess of the sigmoid colon, penetrating to the posterior wall of the uterus (Figure 3). Our patient had an uneventful recovery and no problems have been observed over five years of follow-up.

## Discussion

Among diverticulitis complications, fistula formation may complicate up to 20% of the observed cases. The urinary bladder is the most commonly involved organ. The uterus represents is rarely involved [2]. In 1929, Noecker was the first to report a colouterine fistula secondary to diverticulitis [4]. Inflammatory adherences of the bowel wall to the uterus can occur during acute episodes of diverticulitis, resulting in necrosis and subsequent fistula formation. Fistulae may also develop after localized perforations of diverticula and development of a pericolic abscess [5]. The fact that a sigmoid uterine fistula rarely occurs is thought to be related to the extreme thickness of the uterine wall. Penetration of the sigmoid colon to the wall of the uterus is considered an early stage condition before the formation of a colouterine fistula secondary to diverticulitis.

Our patient demonstrated a relapsing fever but with the symptoms of diverticulitis, such as abdominal pain and less obvious tenderness. In the findings from the gastrograffin enema and colonoscopy, the ring-shaped lesion was revealed as a giant diverticulum of the sigmoid colon without evidence of malignancy, but penetration was not detected. CT and MRI played an important role in the pre-operative surgical planning by demonstrating the extent and degree of pericolonic inflammation. Though identifying any penetration is important for the planning of appropriate surgical management, in this study, neither the imaging nor the colon examinations could detect the penetration.

## Conclusion

We report the case of a patient with penetration of the colon to the wall of the uterus secondary to diverticulitis, together with a relapsing fever. In our patient, pre-operative diagnosis of the penetration was impossible on any imaging and colon examinations. Therefore, in cases of diverticulitis with relapsing fever, and even where no typical symptoms of diverticulitis are present, surgical management should be recommended bearing in mind the possible complication of penetration to other organs.

## Abbreviations

CT: computed tomography; MRI: magnetic resonance imaging.

## Competing interests

The authors declare that they have no competing interests.

## Consent

Written informed consent was obtained from the patient for publication of this case report and any accompanying images. A copy of the written consent is available for review by the Editor-in-Chief of this journal.

## Authors' contributions

TA, YA, NK, KK and TS analyzed the upper endoscopies, collected the clinical data and wrote the manuscript, with contributions from MI. HI, HE, KF, MY, HK and HT collected the clinical data. SY and YR performed the pathological assessment. TA, MI and AN analyzed the endoscopies and participated in the design and coordination of the case report. All authors read and approved the final manuscript.
